# Resveratrol-Coated Balloon Catheters in Porcine Coronary and Peripheral Arteries

**DOI:** 10.3390/ijms20092285

**Published:** 2019-05-09

**Authors:** Stefanie Kamann, Tobias Haase, Nicola Stolzenburg, Melanie Löchel, Daniel Peters, Jörg Schnorr

**Affiliations:** 1 Department of Radiology, Experimental Radiology, Charité—Universitätsmedizin Berlin, 10117 Berlin, Germany; tobias.haase@charite.de (T.H.); Nicola.Stolzenburg@charite.de (N.S.); Melanie.loechel@charite.de (M.L.); Joerg.schnorr@charie.de (J.S.); 2 InnoRa GmbH, 10115 Berlin, Germany; Daniel.peters@innora.de

**Keywords:** drug-coated balloon catheter, restenosis, vascular healing, resveratrol, neovascularization

## Abstract

Angioplasty aiming at vascular dilatation causes endothelial denudation and induces complex inflammatory responses that affect vascular healing, including delayed reendothelialization and excessive neointima proliferation. Resveratrol is known for multiple beneficial effects on the vessel wall after systemic treatment or sustained release from a stent. It is also used as an additive on drug-coated balloon catheters (DCB). In this study, the effect of a single dose of resveratrol, three days to four weeks after administration as a balloon coating during angioplasty, was investigated. Sixteen pigs underwent angioplasty with resveratrol-coated or uncoated balloon catheters in coronary and peripheral arteries. Vessels were overstretched by approximately 20% to enhance vessel wall injury and to produce persistent vessel wall irritation. A significantly reduced number of micro vessels and macrophages in the adventitia, as well as an improved reendothelialization of the vessel lumen, were observed in resveratrol-treated peripheral arteries. The coronaries had a much higher injury score compared to peripheral vessels. Resveratrol-dependent reduction of macrophages, micro vessels or acceleration of reendothelialization was not evident in the coronary vessels. Additionally, no significant effect on neointima proliferation and inflammation score in either vessel territory was observed as a result of resveratrol treatment. In conclusion, the results suggest that resveratrol diminishes the inflammatory response and promotes vascular healing in peripheral arteries. These same effects are absent in more severely injured coronary arteries.

## 1. Introduction

Balloon angioplasty is a widely used endovascular standard procedure to widen or reopen vessel obstructions to restore blood flow. Pressure-resistant folded balloons are introduced into the narrowed vessel segment and inflated to a predetermined diameter with a diluted contrast agent at high pressure, thus restoring the original vessel lumen and blood flow. Vessel wall stretch and denudation caused by angioplasty induces several different biologic processes such as inflammation, vasa vasorum neovascularization, reendothelialization and neointima proliferation, which affect vascular healing. Restenosis, characterized by the transdifferentiation and proliferation of vascular smooth muscle cells (VSMCs) and excessive secretion of the extracellular matrix, is one of the most common complications after angioplasty procedures. Cytostatic drugs, such as paclitaxel, are the current “gold standard” in restenosis prophylaxis and target VSMC proliferation. The use of cytostatics as a stent or balloon coating enables targeted drug delivery, preventing the side effects of systemic administration. However, the antiproliferative effects of cytostatics do not only act on VSMCs but also on endothelial cells (EC), leading to delayed reendothelialization, prolonged vascular healing and an increased risk of thrombosis [1,2,3]. Consequently, there is a need for therapies that not only reduce the risk of restenosis, but also accelerate reendothelialization.

Resveratrol, a grape polyphenol, is used as an antioxidative additive on paclitaxel-coated balloon catheters [4,5,6]. Numerous studies have indicated positive effects of resveratrol on cardiovascular diseases. Continuous systemic resveratrol treatment reduces intimal hyperplasia and improves reendothelialization after vascular injury in several animal models [7,8,9,10]. Local resveratrol treatment has been tested. Results indicate beneficial effects. Implantation of resveratrol-releasing stents in rats resulted in reduced intimal hyperplasia and improved reendothelialization [11]. Furthermore, use of a perforated balloon catheter delivering a resveratrol solution led to reduced neointima proliferation after injury to rabbit iliac arteries [12]. However, results are conflicting, as in the same study the in vivo use of the carrier solution without resveratrol had similar antiproliferative effects, suggesting that this is not exclusively due to resveratrol but possibly to the high content of Kolliphor in the carrier solution [12].

Enhancement of the duration of action and bioavailability of resveratrol is currently under development. Recently, a resveratrol-eluting nanoparticle system for a sustained release of locally-administered resveratrol and a resveratrol–lipid conjugate to enhance the bioavailability of resveratrol were tested in vitro [13,14]. However, the benefits on vascular healing using a local single-dose administration of resveratrol as a balloon coating has not yet been tested.

In the current study, we focus on the use of resveratrol as a dry balloon catheter coating. A porcine model is used to investigate the effect of a local single-dose resveratrol treatment on coronary and peripheral arteries. In Speck et al. [4], the first data on the drug distribution and pharmacology of paclitaxel/resveratrol-coated balloon catheters were recently published. In vivo data on resveratrol-only coated balloons indicate positive effects of resveratrol on the vessel wall of peripheral arteries, without affecting neointima proliferation. In the current study, we investigate resveratrol effects on coronary and peripheral arteries at defined time points.

## 2. Results

### 2.1. Histological Analysis

#### 2.1.1. Injury Score and Morphometry

**Figure A1:**
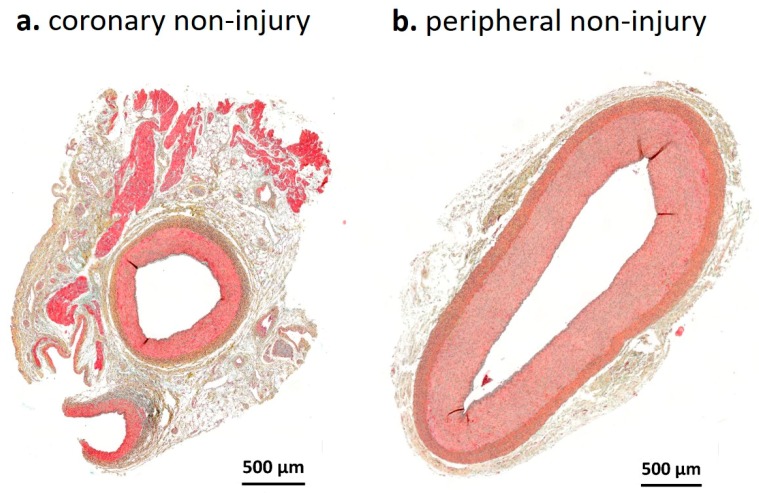
Movat pentachrome-stained cross sections of representative non-injured coronary (**a**) and peripheral (**b**) arteries.

Injury scores reflect damage due to overstretch, unrelated to balloon coating. Scores in the coronary arteries were significantly higher than in peripheral arteries (*p* < 0.0001). Numerous dissections and ruptures of the internal elastic lamina (IEL) could be observed in the coronaries, while peripheral arteries appeared only slightly injured. Sham control arteries are shown in Figure A1.

**Figure 1:**
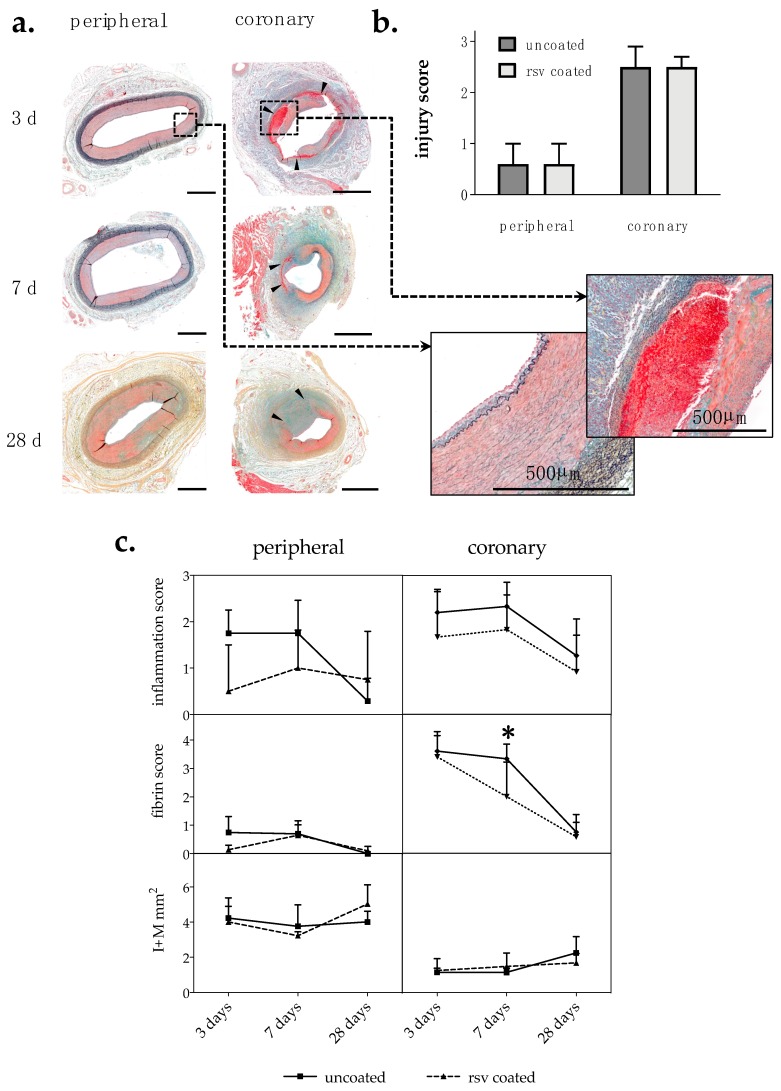
(**a**) Movat pentachrome-stained cross sections of representative coronary and peripheral arteries 3 days, 7 days and 28 days post injury treated with uncoated balloon catheters. Blue: Ground substance and degenerated tissue; red: Muscle; bright red: Fibrin; black: Elastic fiber. Bar = 1 mm. Arrowheads indicate severe injury with media rupture and dissection. (**b**) Injury score of peripheral and coronary arteries; *n* ≥ 22 coronary; *n* ≥ 15 peripheral. (**c**) Histological and morphometric analyses of Movat pentachrome-stained vessel sections. One cross section each from three section levels per vessel were analyzed. I + M: Intima–media area. rsv: resveratrol. *n* ≥ 4 peripheral; *n* ≥ 5 coronary; *n*: Number of arteries; values are mean ± SD; *p*-value determined by Mann–Whitney test; * *p* ≤ 0.05.

To assess vascular remodeling, histomorphometric analysis of treated vessels was performed at 3, 7 and 28 days post intervention. One artery cross section each from three cutting levels per vessel segment were analyzed. As a result of strong vessel injury and frequent rupture of the IEL, absolute media and neointima area could not be determined in coronary arteries. Therefore, the combined intima–media area (I + M) was calculated to quantify neointima proliferation. Different coronary artery types (left anterior descending (LAD), left circumflex coronary (LCX) and right coronary artery (RCA)) were summarized into one coronary group for statistical analysis. Overall, our in vivo balloon injury model rarely led to neointima formation up to 28 days. Furthermore, resveratrol treatment had no influence on neointima proliferation, as seen in Figure 1c and Table 1.

#### 2.1.2. Inflammation Score and Fibrin Score

As a result of vascular injury, inflammatory cells migrate from the vessel lumen and the surrounding tissue into the adventitia. Semi-quantitative analysis of inflammation was done by scoring the number of infiltrated inflammatory cells in sections of the vessel wall. In general, we found the maximum inflammatory cell infiltration within the first 7 days post angioplasty, which then declined. The lowest inflammation scores were evident in the resveratrol-treated peripheral vessel segments. However, no statistically significant differences were found between the treatment groups, as seen in Figure 1c and Table 1. 

Semi-quantitative analysis of fibrin deposition was used as an additional parameter for inflammation and delayed vascular healing [15]. Fibrin was mainly observed in regions with severe injury, e.g., IEL rupture, media rupture or dissection. Depending on the observed strength of injury, there was stronger fibrin deposition in coronary arteries than in peripheral arteries. The maximum fibrin deposition was observed within the first week post injury (p.i.) in all tested vessel segments and then declined. In the coronaries, fibrin drastically decreased from day 3 to day 7 and day 28 post intervention. Resveratrol treatment significantly accelerated this decline in fibrin content in the coronaries by 40 ± 37% at day 7 post angioplasty, as seen in Figure 1a,c and Table 1.

### 2.2. Immunofluorescent Analyses

#### 2.2.1. Vasa Vasorum Neovascularization

**Figure 2:**
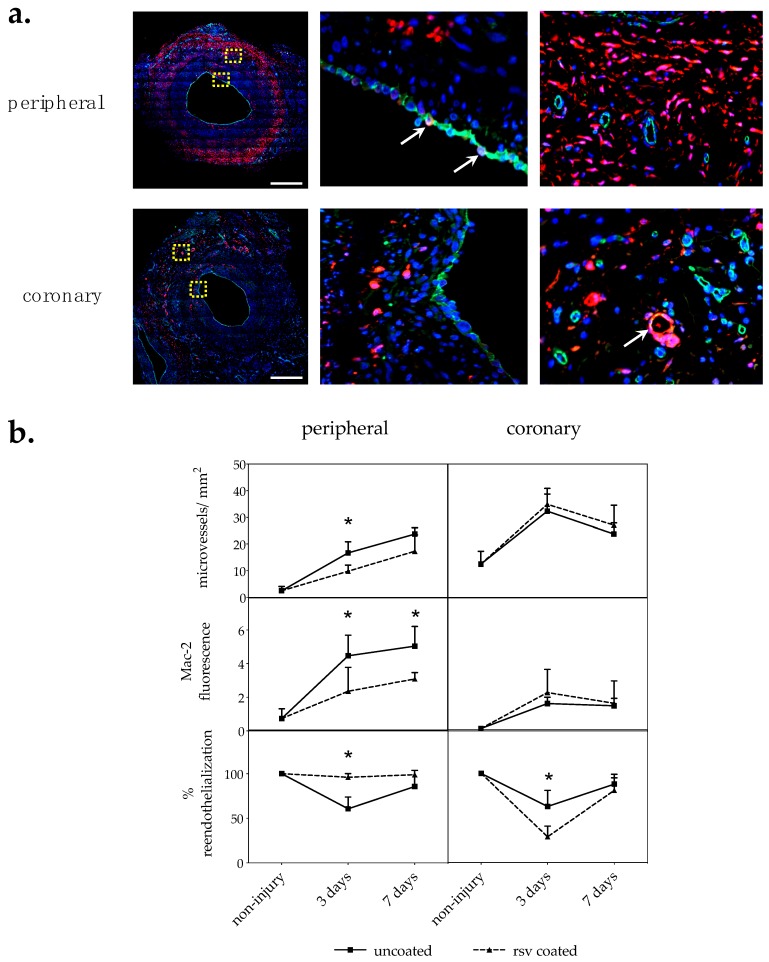
(**a**) Immunofluorescence. CD31/Mac-2 double-stained cross sections of representative coronary and peripheral arteries 7 days post injury treated with uncoated balloon catheters. Red: Mac-2; green: CD31, blue: DAPI. Yellow boxes are shown enlarged next to the picture. Arrows indicate double positive cells. Bar = 0.5 mm. (**b**) Immunohistological analysis. CD31/Mac-2 double staining. Two sections from at least two section levels per vessel were analyzed. *n*: Number of arteries; *n* = 4 peripheral; *n* ≥ 5 coronary; values are mean ± SD; *p*-value determined by Mann–Whitney test; * *p* ≤ 0.05.

The vasa vasorum is a dynamic system of micro vessels surrounding arteries with lumen diameters of more than 0.5 mm that modulates its density in response to the pathophysiological state of the artery. Hence, we counted the number of micro vessels on CD31/PECAM–1-stained cross sections per area in the adventitia. In general, more vasa vasorum micro vessels could be found in coronary than in peripheral arteries, which is in accordance with previous studies [16,17]. In peripheral arteries, treatment with resveratrol-coated balloon catheters reduced the number of micro vessels per mm^2^ in the adventitia 3 days p.i. by 41.1 ± 13.7% (*p* = 0.029) compared to control vessels treated with uncoated balloon catheters. In the coronary arteries, resveratrol treatment had no statistically significant effect on vasa vasorum density, as seen in Figure 2b and Table 1.

#### 2.2.2. Macrophages

Macrophage infiltration is a characteristic biologic process involved in vascular healing after percutaneous transluminal interventions. The macrophage cell surface molecule Mac-2/galectin3 is used as a general inflammation marker [18]. Thus, Mac-2 relative fluorescence was measured as mean value per section. Two sections from at least two section levels per vessel segment were analyzed. In the peripheral vessels, resveratrol significantly reduced Mac-2-positive cells 3 days p.i. by 47.3 ± 31.8% (*p* = 0.013) and 7 days p.i. by 38.6 ± 7.5% (*p* = 0.029) compared to the uncoated control group, as seen in Figure 2b and Table 1. In the coronaries, no differences between the treatment groups could be detected.

#### 2.2.3. Reendothelialization

Reendothelialization was evaluated by labeling CD31/PECAM-positive cells outlining the lumen. The proportion of lumen circumference covered by CD31 cells was estimated. Three days after angioplasty with uncoated balloon catheters, only 60–63% lumen circumference was covered with endothelial cells. Surprisingly, the effect of resveratrol coating seemed to be contradictory in the peripheral and coronary arteries. Resveratrol significantly accelerated reendothelialization of the vessel lumen in peripheral arteries by 54 ± 19% (*p* = 0.029), while reendothelialization in the coronaries was delayed by 58 ± 7% (*p* = 0.019) 3 days p.i., as seen in Figure 2b and Table 1.

Interestingly, cells outlining the vessel lumen occasionally appeared double stained, indicating that these cells express the macrophage marker Mac-2/galectin3 as well as the endothelial adhesion molecule CD31/PECAM-1 on the cell surface, see Figure 2a.

## 3. Discussion

Vessel overstretch and endothelial denudation as a result of balloon angioplasty induce a cascade of biological processes involved in vascular healing and remodeling. The beneficial effects on vascular healing in porcine peripheral arteries from resveratrol dry-coated balloon catheters was demonstrated in this study. The same effect was not observed in coronary arteries.

In the course of the study we found that vessel injury scores in the peripheral arteries were significantly lower than that in the coronary arteries. The difference in the protective effect of resveratrol between arteries may also be explained by the different injury levels.

We focused on analyzing resveratrol-dependent effects in porcine vessels at 3 and 7 days post balloon treatment. Local resveratrol treatment significantly reduced vasa vasorum neovascularization in peripheral arteries 3 days post injury. The vasa vasorum is an adventitial micro vessel system that dynamically adapts its density according to the pathophysiological state of the artery [19,20]. Adventitial neovascularization occurs in response to balloon injury [19] to provide nutrients and oxygen but also contributes to increased leucocyte infiltration [21]. It has been demonstrated that the prevention of vasa vasorum neovascularization reduces neointima formation [22] and macrophage accumulation [21].

In line with these findings, our analysis showed that resveratrol treatment significantly reduced the number of macrophages in the adventitia and perivascular space in the peripheral arteries. However, this effect was not observed in coronary arteries.

Interestingly, resveratrol improved reendothelialization in the peripheral arteries and delayed reendothelialization in the coronaries. We assume that this is due to the stronger vessel injury in the coronaries in combination with the rough balloon surface of the high dose resveratrol-coated balloons, leading to lower initial endothelialization values compared to the uncoated balloons. Previous studies demonstrated that continuous systemic-administered resveratrol improves endothelial wound healing [8]. 

Another parameter that is associated with delayed vascular healing after stent implantation and paclitaxel treatment is prolonged fibrin deposition [15]. In early phases of wound healing, fibrin deposition is considered a first step in repair, i.e., the formation of a “provisional” extracellular matrix [23,24]. Mostly, fibrin depositions are observed in vessels with severe injury including media rupture and dissections. The maximum amount of fibrin was found at 3 days post intervention and then declined within 4 weeks. No significant difference in fibrin deposition was observed between treatment groups and time points.

In contrast to previous findings [9,10,11,12,25], resveratrol treatment in this study had positive effects on vascular healing without affecting neointima proliferation. The results suggest that this could be a result of the limited induction of neointima proliferation in porcine vessels following balloon angioplasty without stent implantation. However, another factor could be the single administration and/or drug dose of resveratrol. 

In a prior pharmacokinetic experiment on porcine coronary arteries, we found that 94% of the resveratrol coating was released during angioplasty and 3% of the initial resveratrol dose could be detected in the treated artery segment. The high water solubility of resveratrol could be one attributing factor, and the rapid metabolic conversion of resveratrol into glucuronide and sulfate derivatives [12,26] could be another factor. Finally, the direct interaction of resveratrol with proteins such as sirtuins [27] also results in a very short persistence of free resveratrol in the tissue.

In general, our findings support the current literature showing the positive effects of resveratrol by stimulating vessel wall healing after balloon injury. However, the results indicate that these effects are modest and likely do not overcome inflammatory processes after severe vascular damage. We assume that this is due to the limited drug dose on the balloon surface and the relatively short duration time that determines local resveratrol dosage.

In summary, we have shown that a local single-dose resveratrol treatment has positive effects on vascular healing in moderately injured peripheral arteries after balloon angioplasty. This includes reduced adventitial neovascularization and macrophage accumulation, as well as accelerated reendothelialization.

Further studies are necessary to clarify if the positive effects of single resveratrol administration on vascular healing, demonstrated in this study, can counteract cytostatic-induced delay in vascular healing and reendothelialization. Hence, investigation of resveratrol in combination with a cytostatic coating would be in accordance with actual practice.

## 4. Materials and Methods 

### 4.1. Angioplasty Balloon Catheters and Coating Procedure

The balloon catheters used in this study were Falcon Bravo 3.5–20 mm, Rx 0.014“, 145 cm shaft for coronary arteries and ClearStream PSC2 6.0–60 mm or 7.0–60 mm, OTW 0.035“ for peripheral arteries. Balloons of these catheters were coated with 6.1 ± 0.2 µg/mm² resveratrol. The coating solution contained 25 mg/mL resveratrol in tetrahydrofuran/acetone/aqua (50/25/25 (m/m)). The coating procedure was done with a Hamilton syringe on semi-expanded balloons with automatic rotation. The balloons were then folded back and sterilized by DMB Apparatebau, Wörrstadt, Germany; 38 °C/6% ethylene oxide/240 min.

### 4.2. Animal Experiments

All animal studies were conducted at the Institute of Medical Technology and Research (IMTR), Rottmersleben, in accordance with the guidelines of the commission directive 86/609/EEC and the German Animal Protection Act based upon the Animal Ethics Committee approvals, IMTR 42502-2-1226, Sachsen–Anhalt, Germany. Date of approval 25 November 2013.

Sixteen domestic pigs, 3 months old, with body weights between 24.0 and 30.5 kg, underwent balloon angioplasty in three coronary and two peripheral arteries each with uncoated and resveratrol-coated balloon catheters.

Details were described previously [28]. Briefly, 2 days before treatment, anticoagulants (75 mg Clopidogrel and 100 mg acetylsalicylic acid) were administered. Long-acting Verapamil was given within 24 h prior to the procedure to reduce vascular spasm during the procedure. The pigs were sedated before general anesthesia was induced. Blood pressure was recorded before and after the treatment. Throughout the procedure, the electrocardiogram, arterial oxygen saturation (SpO2) and temperature were monitored. Access was provided through an external carotid artery. Heparin 5000 IU and 250 mg lysine acetylsalicylate were administered intra-arterially. Vessel segments in coronary or iliac/femoral arteries were selected. The balloons were deployed as indicated and inflated for 60 s with 8 to 14 atm to achieve a balloon-to-artery diameter ratio of approximately 1.2. Pigs were sacrificed at 3 days, 7 days or 28 days after balloon treatment. For euthanasia, 10 mL super-saturated potassium chloride (25%) was injected intravenously in deep anesthesia. 

### 4.3. Histochemistry and Morphometry

Treated vessel segments were dissected, fixed in 4% formalin solution for 24 h and embedded in paraffin. Each segment was cut in three levels. Three sections of each level were stained by Movat pentachrome staining for histomorphometric analysis, see Table A1. Microscopic images were acquired with an AxioObserver.Z1 (Carl Zeiss Vision GmbH, Jena, Germany) and analyzed using Image J software (National Institutes of Health, Bethesda, MD, USA). Due to frequent IEL rupture in the coronaries, neointima formation was quantified by the intima–media area (I + M) instead of the intima/media ratio (I/M).

We defined injury score, inflammation score and fibrin score for balloon-treated vessels without stent based on a previously described injury score [29], and an inflammation score [30] for stent treated vessels as follows. Injury score: (0) IEL intact with endothelium typically denuded, (1) IEL lacerated, (2) IEL lacerated with media visibly lacerated but external elastic lamina (EEL) intact, (3) IEL lacerated with media visibly lacerated, EEL lacerated and/or dissections. Inflammation score: (0) no inflammatory cells, (1) light to moderate number of inflammatory cells, (2) moderate to strong infiltration of inflammatory cells, (3) strong infiltration of inflammatory cells in the quadrant. Fibrin score: (0) no fibrin deposition, (1) light fibrin deposition, (2) moderate fibrin deposition, (3) moderate to strong fibrin deposition, (4) strong fibrin deposition in the quadrant. All scores were assessed semi-quantitatively for each section quadrant. The worst value per cross section was used for further calculation.

### 4.4. Immunohistochemistry

Double immunofluorescent staining of Mac-2 (clone M3/38, Cedarlane, Burlington, Ontario, ON, Canada) and CD31/PECAM-1 (clone M-20-R, Santa Cruz Biotechnology, Dallas, Texas, TX, USA) was done to determine neovascularization, infiltration of macrophages into the vessel wall and reendothelialization of the vessel lumen.

Sections were deparaffinized and antigen retrieval was conducted by boiling in citrate buffer pH 6 for 30 min. Sections were incubated with primary antibodies (1:100) in Dako REAL antibody diluent (Agilent, Santa Clara, California, CA, USA) at 4 °C overnight followed by the incubation of AlexaFluor-labeled secondary antibodies (Invitrogen, Carlsbad, California, CA, USA), 1:200, 45 min at room temperature.

Microscopic images were acquired with an AxioObserver.Z1 and an Apotome (Carl Zeiss Vision GmbH, Jena, Germany) and analyzed using Image J software (National Institutes of Health, Bethesda, MD, USA). Two sections from at least two levels per vessel segment were stained and analyzed. The relative fluorescence of Mac-2-labeled cells was quantified. Reendothelialization was determined by labeling CD31-positive cells outlining the lumen and by the measurement of the lumen circumference covered by CD31 cells in increments of 5%. Neovascularization was analyzed by counting CD31-labeled micro vessels in the adventitia per area.

### 4.5. Statistical Analysis

Quantitative and semi-quantitative parameters were compared by Kruskall–Wallis tests for nonparametric values followed by Mann–Whitney tests to determine statistical significance between treatment groups at each time point using Prism 8 (GraphPad Software, San Diego, California, CA, USA). A *p*-value < 0.05 was considered statistically significant. Data are presented as the mean value ± SD.

## Figures and Tables

**Table 1:** Summary of results. *n*-value: Number of arteries included; I + M: Intima–media area; Macrophages: Relative fluorescence of immunofluorescent-stained Mac-2; values are mean ± SD; *p*-value determined by Mann–Whitney test; * *p* ≤ 0.05.

Survival	Analysis Parameter	Peripheral		*p*-Value	Coronary ^1^		*p*-Value
		Uncoated	rsv-Coated		Uncoated	rsv-Coated	
3 days	*n*-value	4	4		6	5	
	Injury score	1.00 ± 1.41	0.00 ± 0.00		3.00 ± 0.00	2.60 ± 0.55	
	Inflammation score	1.75 ± 0.50	0.50 ± 1.00	0.143	2.20 ± 0.45	1.67 ± 1.03	0.515
	Fibrin score	2.00 ± 0.82	0.75 ± 0.50	0.114	3.60 ± 0.55	3.40 ± 0.89	0.999
	I + M (mm^2^)	4.23 ± 1.15	4.01 ± 0.89	0.999	1.15 ± 0.23	1.25 ± 0.67	0.999
	Macrophages	4.47 ± 1.22	2.36 ± 1.42	0.013 *	1.62 ± 0.38	2.28 ± 1.38	0.400
	% Reendothelialization	61 ± 13	96 ± 4	0.029 *	63 ± 18	29 ± 12	0.019 *
	Neovascularization	16.67 ± 4.21	9.82 ± 2.28	0.029 *	38.79 ± 7.73	41.92 ± 7.14	0.792
7 days	*n*-value	4	4		6	5	
	Injury score	1.00 ± 1.41	0.25 ± 0.50		3.00 ± 0.00	2.20 ± 1.30	
	Inflammation score	1.75 ± 0.71	1.00 ± 0.82	0.486	2.33 ± 0.52	1.83 ± 0.75	0.394
	Fibrin score	1.50 ± 1.00	1.50 ± 0.58	0.999	3.33 ± 0.52	2.00 ± 1.22	0.033 *
	I + M (mm^2^)	3.76 ± 1.22	3.23 ± 0.22	0.343	1.14 ± 0.23	1.48 ± 0.76	0.792
	Macrophages	5.04 ± 1.17	3.09 ± 0.38	0.029 *	1.49 ± 0.44	1.64 ± 1.33	0.931
	% Reendothelialization	86 ± 18	99 ± 1	0.114	88 ± 7	81 ± 18	0.879
	Neovascularization	23.77 ± 2.38	17.33 ± 6.12	0.114	28.51 ± 4.99	32.52 ± 9.02	0.537
28 days	*n*-value	7	7		12	12	
	Injury score	0.14 ± 0.38	1.00 ± 1.31		2.08 ± 0.90	2.50 ± 0.52	
	Inflammation score	0.29 ± 0.49	0.75 ± 1.04	0.511	1.27 ± 0.79	0.92 ± 0.79	0.214
	Fibrin score	0.00 ± 0.00	0.50 ± 0.76	0.200	0.75 ± 0.62	0.58 ± 0.51	0.720
	I + M (mm^2^)	4.01 ± 0.61	5.03 ± 1.09	0.073	2.25 ± 0.92	1.68 ± 0.53	0.151

^1^ Data pool of left anterior descending artery (LAD), left circumflex coronary artery (LCX), and right coronary artery (RCA).

## Abbreviations

CD31/PECAM	Cluster of differentiation 31/platelet endothelial cell adhesion molecule
DCB	Drug-coated balloon
DAPI	4′,6-diamidino-2-phenylindole
EC	Endothelial cell
EEL	External elastic lamina
IEL	Internal elastic lamina
I/M	Intima–media ratio
I + M	Intima–media area
LAD	Left anterior descending artery
LCX	Left circumflex coronary artery
Mac-2	Macrophage marker 2 (galactoside-binding protein of macrophages, also known as Galectin 3)
PTX	Paclitaxel
p.i.	Post injury
RCA	Right coronary artery
RSV	Resveratrol
VSMC	Vascular smooth muscle cell

## Appendix A

**Table A1:** Number of animals, arteries and sections included in the analysis. Movat-stained sections were used to analyze injury score, inflammation score, fibrin score and I + M. Mac-2/CD31 double-stained sections were used to analyze macrophages, reendothelialization and neovascularization.

Animals	Survival	Artery Type	Staining Method	Vessels (Uncoated, rsv-Coated)	Section Levels	Sections Per Level	Sections Total
**4**	3 days	peripheral	Movat	4, 4	3	1	24
			Mac-2/CD31	4, 4	3	2	48
		coronary	Movat	5, 5	3	1	30
			Mac-2/CD31	6, 5	3	2	66
**4**	7 days	peripheral	Movat	4, 4	3	1	24
			Mac-2/CD31	4, 4	3	2	48
		coronary	Movat	6, 5	3	1	33
			Mac-2/CD31	6, 5	3	2	66
**8**	28 days	peripheral	Movat	7, 8	3	1	45
		coronary	Movat	11, 12	3	1	69
